# How Does the Latest Clinical Pharmacology Data & Modeling Support Continued Lecanemab Dosing?

**DOI:** 10.1002/alz.092091

**Published:** 2025-01-09

**Authors:** Larisa Reyderman, Brian A. Willis, Natasha Penner, Arnaud Charil, Shobha Dhadda, Steven Hersch, Michael C. Irizarry, Lynn D Kramer

**Affiliations:** ^1^ Eisai Inc., Nutley, NJ USA; ^2^ Deep Human Biology Learning (DHBL), Eisai Inc., Nutley, NJ USA

## Abstract

Lecanemab is a humanized IgG1 monoclonal antibody binding with high affinity to protofibrils of amyloid‐beta (Aβ) protein. In 18‐month clinical studies, lecanemab has been shown to reduce a complex group of protein interactions associated with early symptomatic Alzheimer’s disease (AD) and slow decline on clinical endpoints of cognition and function for up to 30 months to date. In prior research, results from the phase 2 study gap period (no study drug treatment) between the end of the study core and the beginning of retreatment in the open‐label extension (OLE) provides evidence regarding the need for continued maintenance therapy beyond 18 months. Clinical pharmacology data can help supplement the clinical and mechanistic data to establish the rationale for ongoing treatment. Herein, we will present how the latest clinical pharmacology data and modeling support continued long‐term maintenance lecanemab dosing. Data from the lecanemab phase 2 study (Study 201) and Clarity‐AD (Study 301) were pooled, and biomarker, amyloid PET, and CDR‐SB scores were used to develop models describing the change in amyloid PET and plasma biomarkers with lecanemab treatment. A model was also developed to show how change in amyloid PET predicts slowing of disease progression. These models were used to explore the change in plasma biomarkers, amyloid PET, and CDR‐SB over 4 years, and to evaluate the effect of transitioning to less frequent dosing of lecanemab following 18 to 24 months of initial treatment. Simulations projected that CDR‐SB difference between lecanemab and placebo subjects continued increasing over the 4‐year simulation period. Low amyloid and less severe disease at baseline were associated with slower disease progression. Continued treatment with lecanemab at less frequent dosing intervals following an initial treatment period was demonstrated to effectively maintain the benefit associated with lecanemab treatment on plasma biomarkers, amyloid PET, and clinical outcomes as compared to the initial dosing regimen. We will offer learnings from these recent analyses into the ability of amyloid reduction to be predictive of slowing Alzheimer’s disease progress, the importance of early initiation of treatment, and the utility of continued lecanemab maintenance treatment.

